# Complete Blood Count–Derived Inflammation Indices to Predict 3-Year All-Cause Mortality in Patients With Diabetes and Acute Myocardial Infarction in Critical Care: Retrospective Cohort Study With Single-Center External Validation

**DOI:** 10.2196/83328

**Published:** 2026-03-12

**Authors:** Tong Zhou, Kun Yang, Ying Yang, Song-Mei Liu

**Affiliations:** 1 Department of Clinical Laboratory Center for Gene Diagnosis and Program of Clinical Laboratory Zhongnan Hospital of Wuhan University Wuhan, Hubei China; 2 Department of Pharmaceutical Sciences Zhongnan Hospital of Wuhan University Wuhan, Hubei China

**Keywords:** 3-year mortality, acute myocardial infarction, diabetes, inflammation-related indices, machine learning, prognosis

## Abstract

**Background:**

Inflammation plays a pivotal role in the progression of diabetes and its cardiovascular complications, particularly acute myocardial infarction (AMI). Patients with AMI often face high mortality and morbidity, making accurate prognosis crucial for clinical decision-making and outcome improvement.

**Objective:**

This study aims to develop and validate a stacked predictive model using inflammation-related indices to predict 3-year all-cause mortality among patients with severe diabetes who experience acute myocardial infarction (AMI), aiming to improve patient prognosis.

**Methods:**

We included 833 patients with severe diabetes combined with AMI from the Medical Information Mart for Intensive Care IV (MIMIC-IV) database (training/test cohort) and 166 cases from Zhongnan Hospital, China (external validation cohort with 3-year follow-up). A total of 5 inflammation-related indices (lymphocyte-to-monocyte ratio [LMR], neutrophil-to-lymphocyte ratio [NLR], neutrophil-to-platelet ratio [NPR], platelet-to-lymphocyte ratio [PLR], prognostic inflammatory value [PIV]) were analyzed for their association with mortality using Cox proportional hazards models. Kaplan-Meier curves and restricted cubic spline analysis explored survival probabilities and dose-response relationships. A stacked predictive model incorporating these indices was constructed, and its performance was evaluated using the area under the curve.

**Results:**

LMR was a protective factor (hazard ratio [HR] 0.44, 95% CI 0.25-0.77; *P*<.001), while NLR (HR 1.78, 95% CI 1.19-2.65; *P*=.004) and PIV (HR 1.59, 95% CI 1.09-2.30; *P*=.01) were associated with increased mortality risk. Kaplan-Meier analysis showed mortality increased with decreasing LMR quartiles and increasing NLR, NPR, PLR, and PIV quartiles. Restricted cubic spline confirmed that decreasing LMR and increasing NLR, NPR, PLR, and PIV were associated with higher adverse event risk. The predictive model achieved an area under the curve of 0.803 (95% CI 0.736-0.871) in internal testing and 0.781 (95% CI 0.704-0.858) in the external validation cohort.

**Conclusions:**

The stacked predictive model serves as a robust tool for predicting 3-year outcomes in patients with diabetes combined with AMI. Its reliance on routine, low-cost indices highlights its economic viability and potential for widespread clinical implementation, particularly for optimizing resource allocation in resource-limited settings. However, considering the single-center nature of the derivation data, future multicenter validation is essential to verify the model’s generalizability across different health care systems before establishing it as a standard policy for risk stratification.

## Introduction

Diabetes and its complications represent a growing global health challenge, affecting an increasing number of individuals worldwide. According to the International Diabetes Federation, an estimated 537 million individuals were living with diabetes in 2021, and this number is projected to 1.31 billion by 2050, underscoring the urgent need for effective prevention and management strategies [1].

Type 2 diabetes is characterized by systemic inflammation, driven by complex crosstalk between immune cells and β-cells, which plays a pivotal role in disease progression [2]. Importantly, inflammation not only contributes to the pathogenesis of diabetes itself but also plays a critical role in the development of its complications, including cardiovascular diseases (CVDs) such as atherosclerosis and acute myocardial infarction (AMI) [3-5].

CVD remains one of the leading causes of mortality in patients with diabetes, with AMI being one of the most devastating complications [6]. Patients with diabetes not only exhibit a higher incidence of AMI but also experience worse prognoses compared to individuals without diabetes [7]. Hyperglycemia has been implicated in these outcomes by promoting oxidative stress, endothelial dysfunction, and proinflammatory processes, including M1 macrophage polarization and enhanced monocyte adhesion to activated endothelial cells [8]. These inflammatory changes disrupt vascular homeostasis and contribute to microvascular damage [8]. Long-term prognosis in high-risk patients with diabetes combined with AMI plays a critical role in guiding clinical decision-making and improving outcomes. Prognostic studies provide valuable tools for identifying high-risk patients, enabling timely and targeted interventions to reduce mortality and improve survival rates.

Given the pivotal role of inflammation in both diabetes and AMI, there is growing interest in inflammatory markers as prognostic tools. Markers such as the lymphocyte-to-monocyte ratio (LMR), neutrophil-to-platelet ratio (NPR), platelet-to-lymphocyte ratio (PLR), neutrophil-to-lymphocyte ratio (NLR), and prognostic inflammatory value (PIV) have been widely used to reflect systemic inflammation and predict outcomes in CVDs and cancers [9-12]. Importantly, these indices derived from complete blood count (CBC) inherit its advantages of being stable, cost-effective, and widely accessible, making them potentially applicable for routine clinical use and large-scale prognostic studies. However, whether these inflammatory markers could be used for predicting clinical outcomes of patients with diabetes combined with AMI remains unclear. To address this gap, we leveraged data from the Medical Information Mart for Intensive Care IV (MIMIC-IV) database and the Department of Critical Care Medicine, Zhongnan Hospital of Wuhan University, China, to systematically compare and evaluate the prognostic utility of LMR, NPR, PLR, NLR, and PIV in predicting long-term mortality risk and clinical outcomes in this vulnerable population.

## Methods

### Ethical Considerations

This study adheres to the principles declared in the Declaration of Helsinki and STROBE (Strengthening the Reporting of Observational Studies in Epidemiology) guidelines and was approved by the Medical Ethics Committee of Zhongnan Hospital of Wuhan University (approval: 2020009). The Institutional Review Board of Beth Israel Deaconess Medical Center (BIDMC) granted a waiver of informed consent and approved the sharing of research resources. The authors obtained access to the MIMIC-IV database (certificate number: 65461627).

### Source of Data

This study conducted a retrospective analysis using the large, publicly available intensive care database MIMIC-IV (version 3.0).

For external validation, data were obtained from Zhongnan Hospital of Wuhan University, China. This cohort included 166 patients admitted between January 1, 2021, and June 30, 2022. The final follow-up for outcome ascertainment was completed on July 22, 2025, by hospital record review and telephone interviews. During this period, 56 mortality events were recorded.

### Case Inclusion and Exclusion Criteria

Patients diagnosed with both diabetes and AMI were included in this study. Patient identification was based on diagnostic codes from the *ICD-9* (*International Classification of Diseases, Ninth Revision*), and the *ICD-10* (*International Statistical Classification of Diseases, Tenth Revision*). AMI and diabetes mellitus were identified using *ICD-9* and *ICD-10* codes.

The exclusion criteria were as follows: (1) age <18 years; (2) an intensive care unit (ICU) stay of <24 hours; (3) multiple admissions, with only data from the first hospitalization included; and (4) missing data on lymphocyte, monocyte, neutrophil, or platelet count levels.

### Data Extraction

The *psql* tool of PostgreSQL was used to extract the data. Laboratory variables were extracted from the first result recorded within 24 hours of ICU admission. Features with a missing data rate exceeding 30% were excluded, while multiple imputation was applied to those with a missing data rate below 30%. Missing data were handled using multiple imputation by chained equations. We used the predictive mean matching method with 5 imputations (m=5) and set the random seed to 1 for reproducibility. All predictor variables were included in the imputation model.

The LMR was calculated as lymphocyte count (10^9^/L) / monocyte count (10^9^/L), NPR as neutrophil count (10^9^/L) / platelet count (10^9^/L), the PLR as platelet count (10^9^/L) / lymphocyte count (10^9^/L), the NLR as neutrophil count (10^9^/L) / lymphocyte count (10^9^/L), and the PIV as lymphocyte count (10^9^/L) × platelet count (10^9^/L) × monocyte count (10^9^/L)/ lymphocyte count (10^9^/L) [10-12].

### Time-Zero Alignment

All predictor variables (including CBC components and inflammatory indices) were extracted as the first recorded value on admission to prevent any look-ahead bias. To ensure transportability, this identical protocol—using the admission baseline—was applied to the external validation cohort.

### Outcomes

The start date of follow-up was defined as the date of hospital admission, and the study outcome was 3-year mortality.

### Statistical Analysis

As this study is a retrospective analysis, sample size calculations were not performed. For categorical variables, the chi-square test or Fisher exact test was performed for analysis. Student 2-tailed *t* test and the Wilcoxon rank-sum test were used for continuous variables. Kaplan-Meier (K-M) curves and Cox proportional hazards models were used to determine the relationships between LMR, NPR, PLR, NLR, PIV, and the risk of mortality. In Model 1, adjustments were made for age, gender, BMI. In Model 2, further adjustments were made for age, gender, BMI, troponin T, creatine kinase isoenzymes (CK-MB), insulin, history of revascularization (percutaneous coronary intervention and coronary artery bypass grafting), hyperlipidemia, atrial fibrillation, and stroke. Subgroup analysis was performed to examine the relationship between the continuous inflammation index and 3-year mortality across different subgroups. Restricted cubic spline (RCS) analysis was conducted to explore the associations between the 5 indices and mortality in a dose–response manner.

### Feature Selection

The variance inflation factor (VIF) was used to assess multicollinearity among features. Features with a VIF greater than 5 were removed due to concerns about multicollinearity (Table S1 in Multimedia Appendix 1).

To identify the most important features associated with prognosis, the Boruta (version 9.0.0; R packages) algorithm was used. As a robust feature selection method, Boruta identifies all relevant predictors by iteratively comparing the importance of real features with random shadow features. Features marked as important (green) were selected for subsequent machine learning model construction.

### Construction and Validation of Predictive Models

To construct the predictive model, the important features were identified using the Boruta algorithm. Subsequently, the dataset was randomly split into a training set (666/833, 80%) and a testing set (176/833, 20%) with a fixed random seed of 123. Machine learning models were then developed using the H2O AutoML platform (version 3.44.0.3) in R (R Foundation for Statistical Computing). The AutoML process was configured with the following hyperparameters: maximum number of models = 200, maximum runtime = 24 hours, and class balancing enabled (balance_classes = TRUE) to address outcome imbalance. Finally, models were ranked based on the area under the curve (AUC), and a stacked ensemble model was generated using the top-performing base models.

Each base model was trained on prognostic outcomes derived from the training data, and prognostic scores for each patient were calculated and scaled to a range of 0 to 1, with higher scores indicated worse prognosis. An ensemble method was then applied by compiling the top 5 base models, ranked according to their area under the receiver operating characteristic curve, into a matrix of prognostic scores. A second-layer generalized linear model algorithm was then used to create the final model. Five-fold cross-validation was performed during model training to minimize overfitting and ensure the robustness of the results. A probability threshold of .5 was used to classify patients into high- and low-risk groups for the confusion matrix analysis.

Here, 5 inflammation-related indices—LMR, NPR, PLR, NLR, and PIV—were also selected as features for model construction. Separate models were built for each individual feature following the methodology described above. Additionally, a stacked ensemble model incorporating all 5 indices was constructed to explore their combined predictive performance.

## Results

### Baseline Characteristics

**Figure 1:**
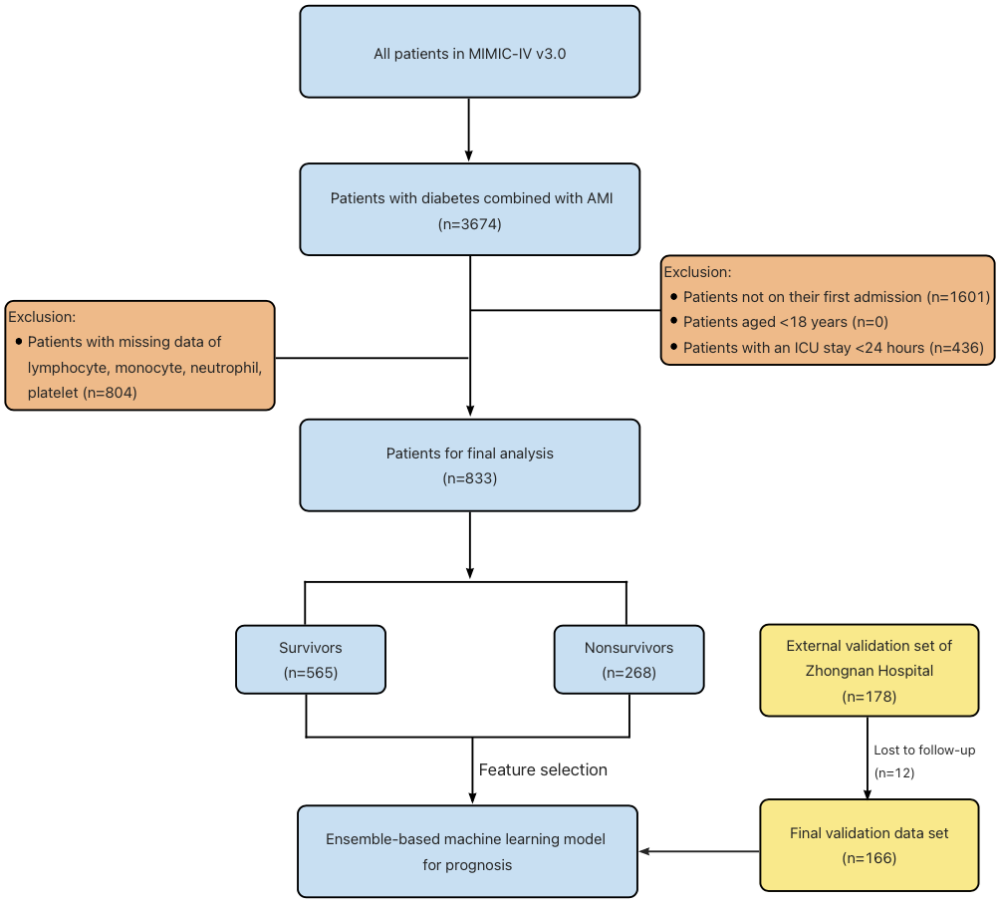
Patient screening and feature selection. Inclusion and exclusion criteria for patients from the Medical Information Mart for Intensive Care IV (MIMIC-IV) database. AMI: acute myocardial infarction; ICU: intensive care unit.

Following the inclusion and exclusion criteria, 833 patients with diabetes combined with AMI from the MIMIC-IV database were used as the training and test cohorts, and 166 cases from Zhongnan Hospital of Wuhan University, China, were enrolled as an external validation cohort. Figure 1 illustrates the flow of patient selection for this study.

Table 1 provides a detailed comparison of baseline characteristics between survivors and nonsurvivors in the MIMIC-IV cohort (n=833), based on the 3-year mortality outcomes. Overall, the nonsurvivors were older and had a lower proportion of males compared with survivors. Among the comorbidities, the nonsurvivors exhibited significantly higher incidences of stroke, atrial fibrillation, and heart failure. Notably, the proportion of patients undergoing coronary artery bypass grafting was significantly lower in the nonsurvivor group. The nonsurvivors also exhibited significant differences in vital signs, including higher heart rate and respiratory rate, as well as higher diastolic blood pressure. As for laboratory parameters, the nonsurvivors had markedly elevated levels of mean corpuscular volume, red cell distribution width, white blood cells, phosphate, potassium, magnesium, alkaline phosphatase, creatinine, and urea nitrogen, suggesting metabolic and immune disturbance. Furthermore, inflammation-related indices also differed significantly between the 2 groups, with nonsurvivors exhibiting higher levels of NPR, PLR, NLR, and PIV, while LMR was lower. However, there was no significant change in troponin T or CK-MB between the survivors and nonsurvivors, demonstrating that these 2 markers typically show a significant increase during the acute phase of myocardial infarction and decrease rapidly during the recovery phase. These findings suggest that CBC-derived inflammation-related indices hold promise for predicting long-term prognosis in patients with diabetes combined with AMI.

**Table 1:** Baseline characteristics between survivors and nonsurvivors.

Characteristics		Total (n=833)	Survivors (n=565)	Nonsurvivors (n=268)	*P* value
**Demographics**					
	Male, n (%)	576 (69.1)	408 (72.5)	168 (62.7)	.07
	Age (years), mean (SD)	68.99 (11.10)	66.92 (10.64)	73.36 (10.81)	<.001
	Weight (kg), mean (SD)	86.65 (23.09)	87.31 (22.07)	85.26 (25.11)	.23
	BMI (kg/m^2^)	29.87 (7.01)	29.85 (6.46)	29.93 (8.06)	.88
**Vital signs, mean (SD)**					
	Heart rate (bpm)	86.82 (18.70)	83.80 (15.87)	93.21 (22.30)	<.001
	Respiratory rate (breaths/min)	18.76 (6.86)	17.50 (5.75)	21.44 (8.14)	<.001
	Systolic blood pressure (mm Hg)	118.67 (23.51)	117.55 (21.82)	120.93 (26.61)	.05
	Diastolic blood pressure (mm Hg)	66.63 (15.75)	65.74 (14.51)	68.49 (17.90)	.02
**Comorbidities, n (%)**					
	Hypertension	293 (35.2)	245 (43.4)	48 (17.9)	<.001
	Stroke	71 (9)	27 (5)	44 (16)	<.001
	Atrial fibrillation	61 (7)	33 (6)	28 (10)	<.001
	Heart failure	457 (54.9)	271 (48)	186 (69.4)	<.001
	Hyperlipidemia	601 (72.1)	438 (77.5)	163 (60.8)	<.001
**Treatment, n (%)**					
	Insulin	809 (97.1)	552 (97.7)	257 (95.9)	.22
	Percutaneous coronary intervention	94 (11)	53 (9)	41 (15)	.02
	Coronary artery bypass grafting	439 (52.7)	396 (70.1)	43 (16)	<.001
**Complete blood count, mean (SD)**					
	White blood cells (10^9^/L)	11.66 (7.42)	10.60 (5.33)	13.89 (10.21)	<.001
	Red cell distribution width (%)	14.41 (2.16)	13.95 (1.76)	15.39 (2.56)	<.001
	Hemoglobin (g/dL)	11.61 (2.36)	12.13 (2.19)	10.51 (2.35)	<.001
	Hematocrit (%)	35.71 (6.69)	37.00 (6.16)	33.01 (6.95)	<.001
	Platelet count (10^9^/L)	216.84 (81.65)	217.75 (77.27)	214.93 (90.31)	.64
	Neutrophil count (10^9^/L)	10.50 (5.78)	9.93 (5.12)	11.70 (6.82)	<.001
	Lymphocyte count (10^9^/L)	1.92 (4.34)	1.98 (1.20)	1.81 (7.46)	.61
	Monocyte count (10^9^/L)	0.78 (0.54)	0.67 (0.48)	0.99 (0.59)	<.001
	Eosinophil count (10^9^/L)	0.13 (0.19)	0.14 (0.18)	0.11 (0.20)	.02
	Basophil Count (10^9^/L)	0.04 (0.04)	0.04 (0.04)	0.04 (0.04)	.25
	Mean corpuscular volume (fL)	90.43 (6.79)	89.84 (6.46)	91.68 (7.30)	.001
	Mean corpuscular hemoglobin (pg)	29.32 (2.56)	29.42 (2.52)	29.11 (2.64)	.10
	Mean corpuscular hemoglobin concentration (g/dL)	32.43 (1.50)	32.74 (1.43)	31.77 (1.43)	<.001
	Immature granulocytes (%)	1.04 (0.92)	1.01 (0.90)	1.10 (0.95)	.17
**Metabolic, mean (SD)**					
	Bicarbonate (mEq/L)	22.44 (4.40)	23.02 (3.94)	21.22 (5.04)	<.001
	Phosphate (mg/dL)	3.95 (1.32)	3.75 (1.15)	4.38 (1.52)	<.001
	Chloride (mEq/L)	101.03 (5.42)	100.99 (4.56)	101.13 (6.89)	.71
	Potassium (mEq/L)	4.38 (0.73)	4.31 (0.65)	4.52 (0.84)	<.001
	Magnesium (mg/dL)	1.98 (0.36)	1.94 (0.34)	2.06 (0.40)	<.001
	Calcium total (mg/dL)	8.70 (0.74)	8.76 (0.69)	8.58 (0.83)	.001
	Aspartate aminotransferase (IU/L)	80.46 (131.84)	69.50 (117.58)	103.85 (155.73)	.001
	Alanine aminotransferase (IU/L)	51.68 (94.95)	42.40 (71.36)	71.30 (129.58)	<.001
	Albumin (g/dL)	3.45 (0.58)	3.61 (0.50)	3.13 (0.61)	<.001
	Alkaline phosphatase (IU/L)	101.09 (104.40)	90.53 (52.22)	123.37 (165.74)	<.001
	Glucose (mg/dL)	203.05 (105.71)	200.08 (102.18)	209.30 (112.72)	.24
	Urea nitrogen (mg/dL)	31.57 (22.82)	25.91 (17.38)	43.49 (27.82)	<.001
	Creatinine (mg/dL)	1.77 (1.68)	1.50 (1.44)	2.33 (1.99)	<.001
	Troponin T (ng/mL)	1.18 (2.57)	1.08 (2.42)	1.39 (2.85)	.10
	Creatine kinase isoenzymes (ng/mL)	28.73 (64.48)	28.48 (60.27)	29.26 (72.68)	.87
**Coagulation, mean (SD)**					
	Partial thromboplastin time (sec)	48.09 (32.14)	48.73 (31.37)	46.73 (33.73)	.40
**Inflammation-related indices, mean (SD)**					
	Lymphocyte-to-monocyte ratio	3.74 (4.28)	4.62 (4.76)	1.93 (2.11)	<.001
	Neutrophil-to-platelet ratio	0.05 (0.05)	0.05 (0.05)	0.06 (0.05)	.001
	Platelet-to-lymphocyte ratio	187.02 (184.20)	164.53 (169.65)	234.37 (203.96)	<.001
	Neutrophil-to-lymphocyte ratio	9.55 (11.84)	7.63 (9.23)	13.61 (15.26)	<.001
	Prognostic inflammatory value	2291.85 (3761.61)	1808.40 (2741.59)	3291.33 (5143.52)	<.001

For the external validation cohort (n=166), baseline analysis focused specifically on CBC data and indices in the prognostic model (LMR, NLR, NPR, PIV, and PLR). The detailed comparison between survivors and nonsurvivors based on the 3-year mortality outcomes are presented in Table S2 in Multimedia Appendix 1.

### Associations Between the Inflammation-Related Indices and Mortality Risk

Cox proportional hazards analysis identified significant associations between inflammation-related indices (LMR, NPR, PLR, NLR, and PIV) and 3-year mortality risk (Table S3 in Multimedia Appendix 1). LMR was determined to be a protective factor, while NPR, PLR, NLR, and PIV were associated with an increased risk of mortality. These findings underscore the prognostic value of systemic inflammation markers in predicting long-term outcomes in this population.

### LMR and Mortality Risk

**Figure 2:**
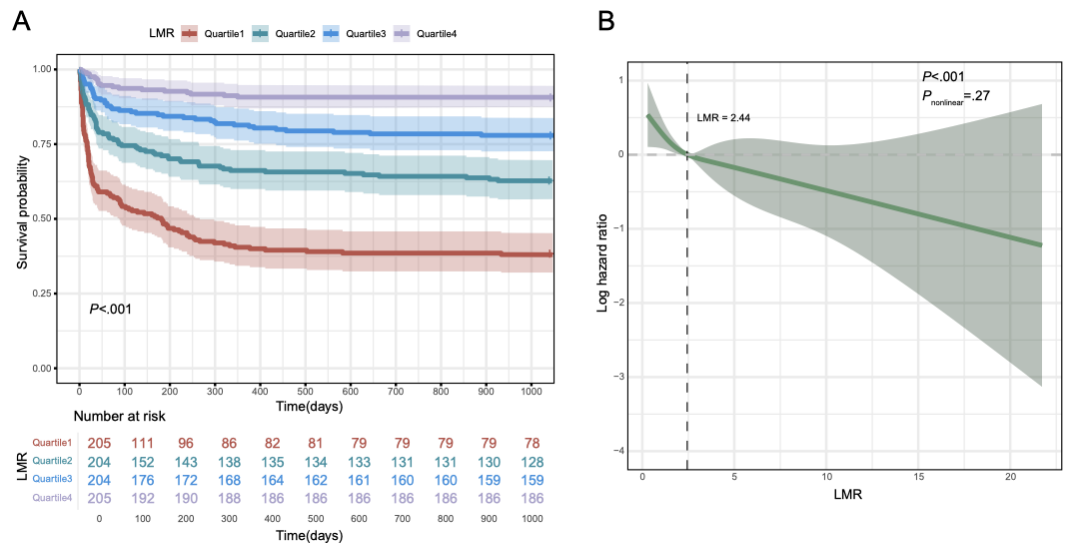
Kaplan‒Meier (K-M) survival analysis curves and restricted cubic spline (RCS) regression analysis of lymphocyte-to-monocyte ratio (LMR) for 3-year mortality. (A) K-M curves of LMR. (B) RCS analysis of LMR.

Patients were stratified into quartiles based on LMR values: quartile 1 (LMR≤1.17; n=205), quartile 2 (1.17<LMR≤2.48; n=204), quartile 3 (2.48<LMR≤4.60; n=204), and quartile 4 (LMR>4.60; n=205). In Cox proportional hazards models, quartiles 2, 3, and 4 were significantly associated with reduced 3-year mortality risk compared to quartile 1, in both unadjusted and adjusted models (Table S3 in Multimedia Appendix 1). K-M survival analysis further demonstrated that patients in quartile 1 had the lowest 3-year survival probability, indicating the detrimental impact of low LMR (Figure 2A). RCS analysis revealed a decreasing trend between LMR and mortality risk, with a critical inflection point at LMR=2.44 (Figure 2B). Below this threshold, lower LMR values were strongly associated with increased mortality risk.

### NPR and Mortality Risk

**Figure 3:**
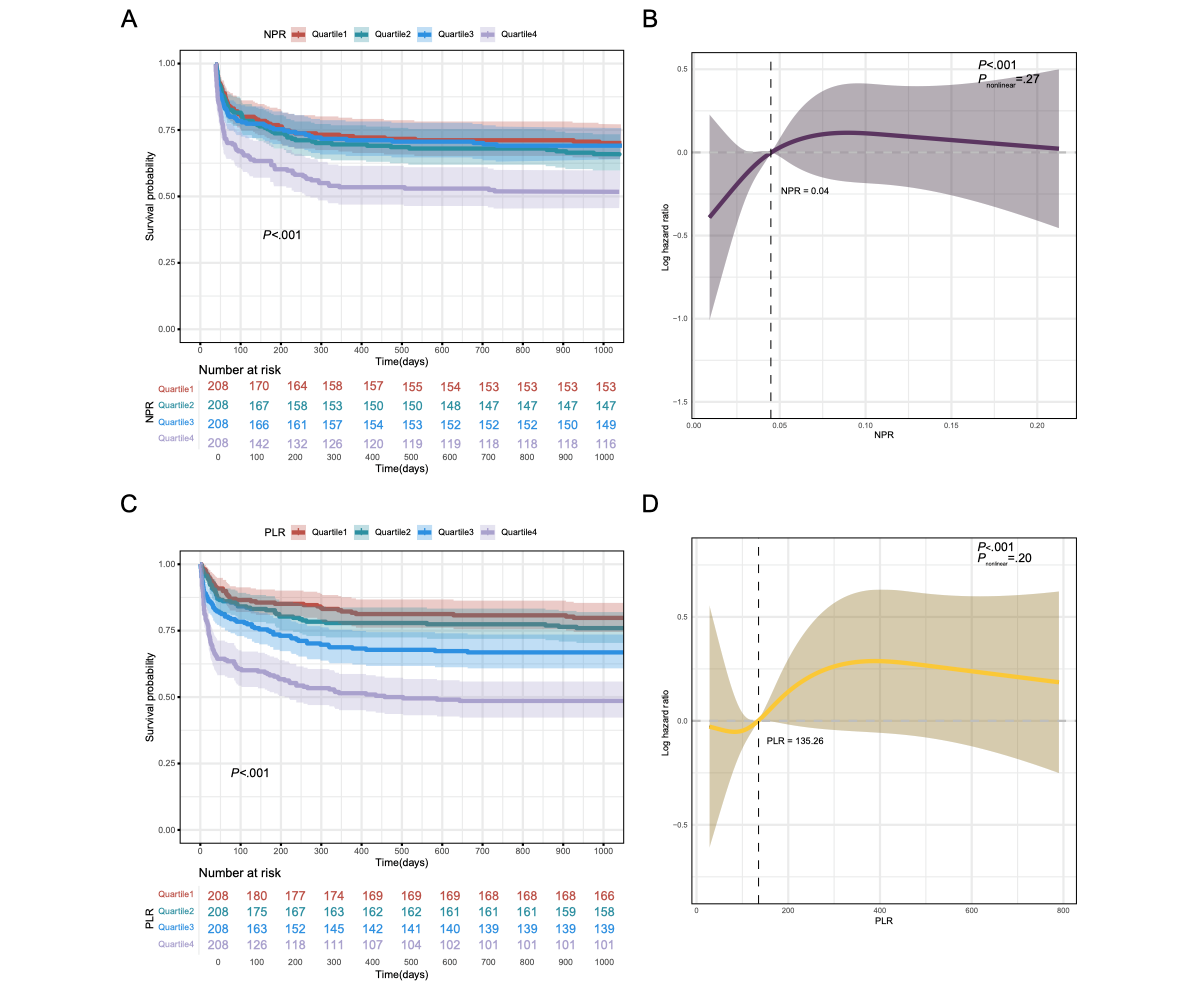
Kaplan-Meier (KM) survival analysis curves and restricted cubic spline (RCS) regression analysis of neutrophil-to-platelet ratio (NPR) and platelet-to-lymphocyte ratio (PLR) for 3-year mortality. (A) K-M curves of NPR. (B) RCS analysis of NPR. (C) K-M curves of PLR. (D) RCS analysis of PLR.

For NPR, patients were divided into 4 quartiles: quartile 1 (NPR≤0.033), quartile 2 (0.033<NPR≤0.046), quartile 3 (0.046<NPR≤0.064), and quartile 4 (NPR>0.064). In the unadjusted model, higher NPR values were significantly associated with increased mortality risk. However, this association was attenuated and lost statistical significance after adjustment for potential confounders (Table S3 in Multimedia Appendix 1). K-M survival analysis demonstrated that patients in quartile 4 (highest NPR) had the lowest 3-year survival probability compared with other quartiles, underscoring the detrimental impact of elevated NPR values (Figure 3A). RCS analysis revealed a nonlinear trend in the association between NPR and mortality risk, with a potential threshold observed around NPR=0.04. However, the test for nonlinearity did not reach statistical significance (*P*_nonlinearity_=.27), suggesting that a linear relationship between these variables cannot be excluded (Figure 3B).

### PLR and Mortality Risk

PLR was categorized into quartile 1 (PLR≤83.50), quartile 2 (83.50<PLR≤134.37), quartile 3 (134.37<PLR≤255.65), and quartile 4 (PLR>255.65). In the unadjusted model, higher PLR values were associated with increased mortality risk. However, after adjustment for confounding variables, this association was no longer statistically significant, suggesting potential confounding effects (Table S3 in Multimedia Appendix 1). K-M survival analysis demonstrated that patients in quartile 4 (highest PLR) had the lowest 3-year survival probability, consistent with the hypothesis that elevated PLR reflects a heightened inflammatory state (Figure 3C). RCS analysis indicated that as PLR increased, mortality risk also increased, with a notable value at PLR=135.26 (Figure 3D).

### NLR and Mortality Risk

**Figure 4:**
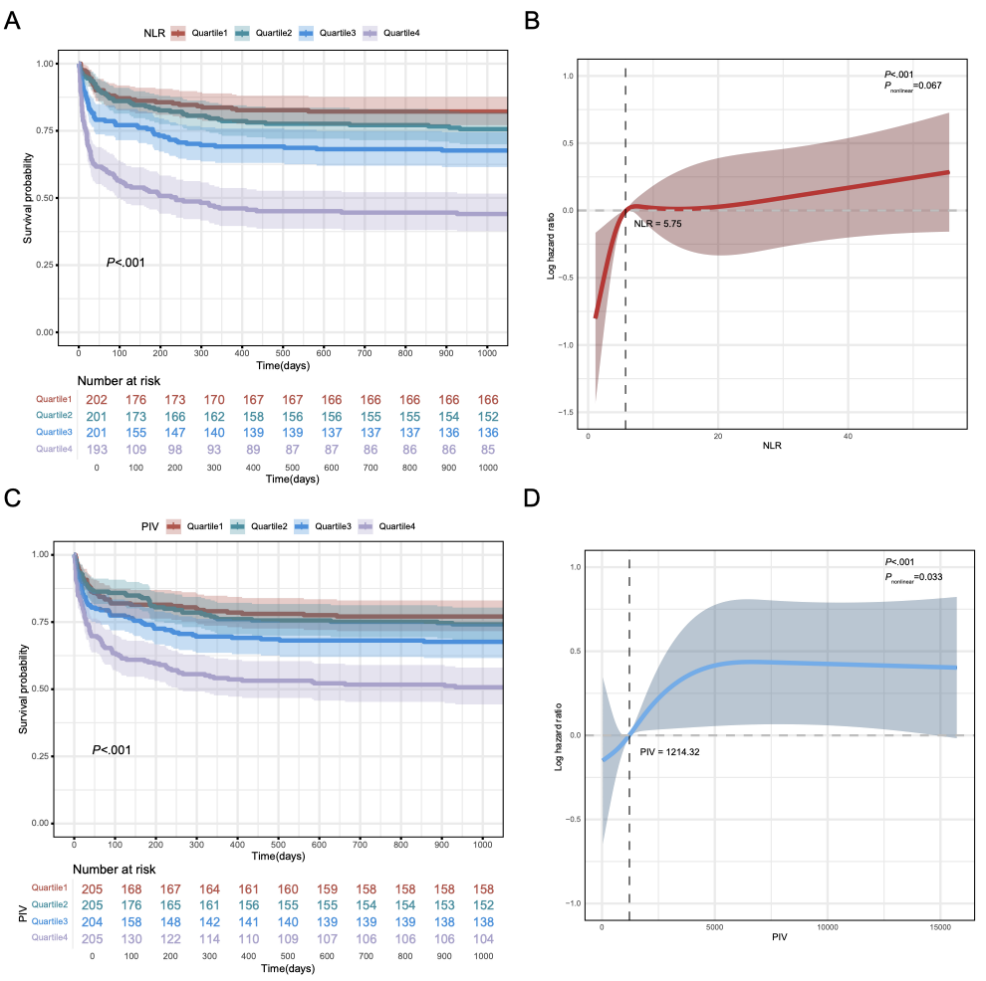
Kaplan-Meier (KM) survival analysis curves and restricted cubic spline (RCS) regression analysis of neutrophil-to-lymphocyte ratio (NLR) and prognostic inflammatory value (PIV) for 3-year mortality. (A) K-M curves of NLR. (B) RCS analysis of NLR. (C) K-M curves of PIV. (D) RCS analysis of PIV.

NLR quartiles were defined as follows: quartile 1 (NLR≤3.50), quartile 2 (3.50<NLR≤5.86), quartile 3 (5.86<NLR≤10.62), and quartile 4 (NLR>10.62). In both unadjusted and adjusted Cox proportional hazards models, patients in quartile 4 (highest NLR) exhibited a significantly increased risk of 3-year mortality compared with those in lower quartiles (Table S3 in Multimedia Appendix 1). K-M survival analysis further demonstrated that quartile 4 had the lowest 3-year survival probability, indicating the adverse prognostic impact of elevated NLR (Figure 4A). RCS analysis indicated that lower NLR values were associated with lower mortality risk, whereas higher NLR values significantly increased mortality risk, especially beyond a threshold of 5.75. Although the *P*=.07 suggests the nonlinear relationship was not fully significant, the trend between NLR and mortality risk is noteworthy (Figure 4B).

### PIV and Mortality Risk

For PIV, the quartiles were defined as quartile 1 (PIV≤550.46), quartile 2 (550.46<PIV≤1207.40), quartile 3 (1207.40<PIV≤2619.32), and quartile 4 (PIV>2619.32). Similarly, in both unadjusted and adjusted Cox models, quartile 4 (highest PIV) was significantly associated with increased 3-year mortality risk compared with lower quartiles (Table S3 in Multimedia Appendix 1). K-M survival analysis demonstrated that patients in quartile 4 had the lowest 3-year survival probability (Figure 4C), suggesting an association between elevated PIV and poor outcomes. The RCS curve shows that 1214.32 is a risk turning point; beyond this value, risk increased significantly (Figure 4D).

### Stratified Analyses

To further explore whether the relationship between inflammation-related indices and 3-year all-cause mortality persists under different conditions, subgroup analyses were conducted based on gender, age, BMI, hypertension, and hyperlipidemia. In the subgroup of patients aged ≥60 years, the hazard ratios (HRs) for all indices related to 3-year all-cause mortality were significant (Figures S1 and S2 in Multimedia Appendix 2). However, in the subgroup of patients aged <60 years, the HRs for NPR, PLR, and PIV did not show statistical significance. Furthermore, the HR for NPR was not statistically significant in the subgroup of patients with BMI<18 kg/m^2^ and the subgroup of patients with hypertension. Similarly, the HRs for NPR and PIV were not statistically significant in the subgroup of patients with BMI>28 kg/m^2^ (Figures S1 and S2 in Multimedia Appendix 2).

### Feature Selection

Initially, we compared the prognostic performance of individual CBC counts (lymphocyte, monocyte, neutrophil, and platelet counts) with inflammation-related indices using Cox regression models. The C-index values for individual cell counts ranged from 0.62 to 0.68, significantly lower than those of composite indices (0.72-0.79; Figure S3 in Multimedia Appendix 2). This disparity highlights the limited utility of isolated cell counts in capturing systemic inflammatory states. Given that the inflammation-related indices are derived from these individual cell counts, they inherently exhibit a higher degree of collinearity. Therefore, to focus more on the inflammation-related features themselves and minimize the impact of collinearity in the analysis, we excluded the individual immune cell counts before performing the VIF calculation.

We then examined the VIF to detect multicollinearity among variables, and features with a VIF>5 were removed (Table S1 in Multimedia Appendix 1). Subsequently, feature selection was performed using the Boruta algorithm, in which variables in the green area were identified as important features, whereas those in the red area were deemed unimportant (Figure S4 in Multimedia Appendix 2). Both CK-MB and troponin T were classified as unimportant (red box in Figure S4 in Multimedia Appendix 2), corroborating their limited prognostic value observed in baseline and Cox analysis (Table 1 and Figure S3 in Multimedia Appendix 2). This exclusion by the algorithm suggests that, although CK-MB and troponin T are critical for diagnosing acute myocardial injury, their transient elevation may not reflect the chronic inflammatory processes that drive long-term mortality in patients with diabetes combined with AMI.

### Development and Validation of an Ensemble Machine Learning Model for Prognostic Prediction

**Figure 5:**
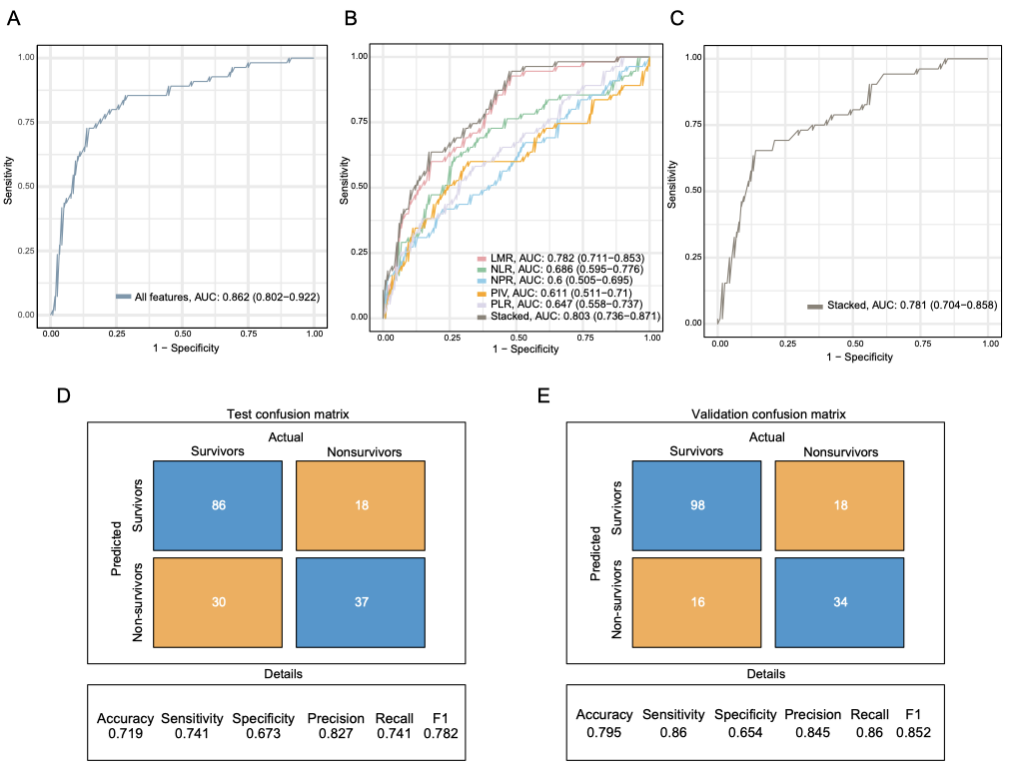
Receiver operating characteristic (ROC) curves of the machine learning prognostic models. (A) ROC curve for the model constructed using all important features in the test cohort. (B) ROC curves for models based on individual inflammation-related indices (lymphocyte-to-monocyte ratio [LMR], neutrophil-to-lymphocyte ratio [NLR], neutrophil-to-platelet ratio [NPR], prognostic inflammatory value [PIV], platelet-to-lymphocyte ratio [PLR]) and their combined model in the test cohort. (C) ROC curve for the combined inflammation-related indices model in the external validation cohort (area under the curve [AUC]=0.781). (D) Confusion matrix for the combined inflammation-related indices model in the test cohort. (E) Confusion matrix for the combined inflammation-related indices model in the external validation cohort.

After feature selection, we constructed a stacked model using all the important features, achieving an AUC of 0.862 in the test cohort (Figure 5A). To further explore the effectiveness of inflammation-related indices in predicting mortality risk in patients with diabetes combined with AMI, we built models based on individual inflammation-related indices, with AUCs of 0.782, 0.686, 0.598, 0.611, and 0.648 for LMR, NLR, NPR, PIV, and PLR, respectively, in the test cohort. We also combined these 5 indices into a stacked model, which achieved an AUC of 0.803 in the test cohort (Figure 5B; detailed parameters are shown in Figure 5D). Importantly, this combined inflammation-related indices model demonstrated robust generalizability, achieving an AUC of 0.781 in the external validation cohort (Figure 5C), with its predictive performance detailed in the confusion matrix (Figure 5E).

Although the combined model performed slightly worse than the model using all important features in the test cohort, it demonstrated comparable and generalizable prognostic ability. The combined inflammation-related indices model may contribute to future prognostic prediction by offering a simplified and cost-effective alternative for clinical applications.

Beyond discrimination, we evaluated the calibration and clinical utility of the model. The calibration plots displayed good agreement between predicted probabilities and observed outcomes, with Brier scores of 0.146 in the validation set and 0.155 in the external cohort (Figure S5 in Multimedia Appendix 2). Furthermore, decision curve analysis (DCA) indicated that the model provided a substantial net benefit over the “treat-all” or “treat-none” strategies across a wide range of threshold probabilities in both cohorts (Figure S5 in Multimedia Appendix 2).

In the external validation cohort, we also evaluated an enhanced model incorporating readily available vitals (age, heart rate, blood pressure, respiration rate). This “Indices + Vitals” model showed marginal improvement (AUC=0.788) and overlapping net benefit curves in DCA (Figure S6 in Multimedia Appendix 2), supporting the use of the 5-index model as a parsimonious stand-alone tool.

We further compared the stacked model with the best-performing single index LMR (Figure S8 in Multimedia Appendix 2). Although the improvements in net reclassification improvement and discrimination improvement were not statistically significant, the stacked model demonstrated superior overall performance. Specifically, it achieved a higher AUC (0.781 vs 0.771 in external validation) and, more importantly, better calibration (Brier score: 0.155 vs 0.183; calibration slope: 1.589 vs 3.147), suggesting that the multi-index approach provides more robust and accurate risk estimates than any single biomarker.

### Application of the Model

Using the above model, we developed a web tool to predict the prognosis of patients with diabetes and acute myocardial infarction (Figure S9 in Multimedia Appendix 2). Users can get prediction results by directly inputting features on the web page [13].

## Discussion

### Overview

To the best of our knowledge, this study is the first to systematically compare 5 inflammation-related indices (LMR, NLR, NPR, PLR, and PIV) derived from CBC in relation to the 3-year mortality risk of patients with diabetes combined with AMI, and to explore their potential application in predicting long-term mortality risk. The findings indicate that inflammation may play a critical role in the long-term prognosis of patients with diabetes combined with AMI. By constructing both single-indicator models and combined predictive models, we demonstrated that these inflammation indices can effectively assess mortality risk while also providing a reference for rapid and straightforward clinical risk assessment methods. Interestingly, adding readily available vital signs to the inflammation-related indices provided limited incremental value in the external cohort, with minimal improvement. This suggests that the 5-index model may offer a streamlined tool for risk stratification (Figure S6 in Multimedia Appendix 2).

From a clinical perspective, the proposed model primarily relies on routine CBC components, which are widely available, rapid, and inexpensive. This accessibility could make the model a potentially useful tool for early risk stratification in settings such as emergency departments and ICUs, potentially helping clinicians to prioritize resource allocation.

The prognostic value of these CBC-derived inflammation-related indices likely stems from the synergistic pathophysiology of diabetes and AMI. The chronic hyperglycemia and insulin resistance unique to patients with diabetes induce low-grade chronic inflammation, which not only exacerbates cardiovascular damage but also drives disease progression through various mechanisms [14]. When AMI occurs, it triggers an acute stress response involving the sympathetic nervous system and the hypothalamic–pituitary–adrenal axis [15]. Both chronic metabolic inflammation and acute ischemic injury leads to a profound dysregulation of hematopoiesis, manifesting as the specific alterations in leukocytes and platelets observed in this study [16].

The elevated risk associated with high NLR, PLR, and PIV can be mechanically explained by the exacerbated proinflammatory and prothrombotic phenotype in patients with diabetes. Hyperglycemia and insulin resistance prime neutrophils to overreact during myocardial ischemia. A critical mechanism linking these cells to poor prognosis is the formation of neutrophil extracellular traps. In a diabetic environment, activated neutrophils release excessive neutrophil extracellular traps, which not only directly damage cardiomyocytes but also serve as a scaffold for platelet aggregation and thrombosis [17,18]. This interaction is particularly detrimental in AMI, as it contributes to microvascular obstruction and extends the infarct size [19]. Furthermore, diabetic platelets exhibit surface protein glycation and increased reactivity, making them more prone to adhering to leukocytes [20]. PIV (the systemic immune-inflammation index), which integrates neutrophils, platelets, and monocytes, essentially captures this extensive interplay between coagulation and inflammation, explaining its strong association with mortality in our cohort of patients with diabetes.

Conversely, the protective role of LMR highlights the importance of adaptive immunity in healing. Lymphocytes, particularly regulatory T cells, are essential for transitioning the immune response from the inflammatory phase to the reparative phase [21]. However, in patients with diabetes complicated by AMI, lymphocytes face dual suppression: chronic oxidative stress from hyperglycemia induces lymphocyte apoptosis, while the acute cortisol surge from AMI causes massive lymphopenia [22,23]. This severe depletion of lymphocytes impairs the containment of the inflammatory storm. Simultaneously, hyperglycemia promotes differentiation of monocytes into the proinflammatory M1 phenotype rather than the reparative M2 phenotype, further delaying scar healing and promoting adverse ventricular remodeling [24,25]. Therefore, a reduced LMR serves as a composite marker indicating an uncontrolled inflammatory response correlating with worse long-term survival.

The elevated levels of NLR, PLR, NPR, and PIV may reflect a proinflammatory state driven by interactions among platelets, neutrophils, and lymphocytes, which could be a critical driver of poor prognosis in patients with diabetes and CVDs. For instance, PIV, as an integrative inflammatory marker, captures the interplay between platelets, neutrophils, and lymphocytes. Studies have shown that elevated PIV levels are associated with worse outcomes in patients with hypertension [20]. Additionally, previous research has demonstrated that high NLR and PLR values are not only independent predictors of in-hospital mortality in patients with heart failure but are also closely associated with the risk of cardiac death [26,27]. Elevated NLR may indicate enhanced proinflammatory neutrophil activity and impaired lymphocyte function, whereas increased PLR may reflect both platelet activation and reduced lymphocyte-mediated immune function. In contrast, NPR primarily reflects the ratio between neutrophils and platelets, and its elevation has been associated with worse outcomes in patients with myocardial infarction with nonobstructive coronary arteries [28].

The protective role of LMR and the risk-associated roles of NLR, NPR, PLR, and PIV further underscore the critical importance of systemic inflammation and immune balance in the prognosis of patients with diabetes combined with AMI. These mechanisms suggest that protective and risk-related inflammatory markers may influence the prognosis of patients with diabetes combined with AMI through distinct pathways, providing a theoretical basis for future research on underlying mechanisms and advancements in diagnostic and therapeutic strategies.

In addition, the findings of this study highlight the significant value of inflammation-related indices in predicting the long-term prognosis of patients with diabetes combined with AMI.

Compared with traditional complex biomarkers or imaging techniques, these inflammation indices are derived from CBC, which is a widely available and affordable diagnostic tool. Furthermore, CBC results are highly stable and reproducible, making them reliable for both research and clinical practice [29]. These advantages highlight their practicality and feasibility for preliminary prognostic assessment in patients with diabetes combined with AMI. The combined predictive model constructed based on these 5 indices (AUC=0.803) demonstrated a predictive ability comparable to that of the full importance features model (AUC=0.862). Moreover, our DCA confirmed that using this model to guide clinical decisions provides a higher net benefit than default strategies across a wide range of threshold probabilities (Figures 5A and B in Multimedia Appendix 2). By identifying high-risk patients, such prognostic models may facilitate early clinical interventions and potentially improve survival rates.

### Limitations

Despite confirming the potential role of inflammation-related indices in predicting the long-term prognosis of patients with diabetes combined with AMI, this study has several limitations.

First, the regulatory mechanisms of inflammation in diabetes and AMI are highly complex, with different types of inflammatory cells potentially exerting bidirectional effects during disease progression. This intricate cellular regulation may not be fully captured by simplified inflammation-related indices.

Second, although 5 commonly used inflammation-related indices were analyzed, other potentially important inflammatory markers (eg, interleukin 6 and tumor necrosis factor α) were not included, which may underestimate the comprehensive impact of inflammation. Additionally, these indices primarily reflect systemic inflammation and may not adequately capture the influence of local immune microenvironments.

Third, a direct comparison with established scoring systems, such as the Global Registry of Acute Coronary Events or the Thrombolysis in Myocardial Infarction score, was not feasible in this study. Critical variables required for these scores—specifically cardiac arrest at admission, Killip classification, and coronary artery stenosis severity—were not systematically recorded or exhibited high levels of missingness in the structured electronic health records of the MIMIC-IV database. Regarding clinical utility, the DCA results suggest that using our model to guide clinical decisions would yield a net benefit over default strategies within a broad threshold range. However, the observed calibration drift in the external validation cohort highlights the heterogeneity between populations (Figures 5A and B in Multimedia Appendix 2). While the model’s discriminative power remains robust, this finding emphasizes the necessity of model recalibration to adjust for baseline risk differences before local clinical implementation.

Fourth, the external validation was asymmetrical. While the primary 5-index model was successfully validated externally, we could not validate the full model containing ancillary covariates because these specific variables were not available in the external center’s dataset. This limitation restricts the generalizability of the covariate-adjusted analysis to the derivation cohort.

Fifth, while MIMIC-IV provides a large-scale, high-quality dataset, it represents a single-center experience in the United States. Consequently, practice patterns, patient demographics, and admission criteria may differ from those in other institutions or countries, potentially limiting the direct generalizability of our findings. Although external validation was performed to assess transportability, future multicenter prospective studies are widely recommended.

Finally, regarding model transparency, while our model demonstrated excellent calibration in the internal validation, calibration drift was observed in the external validation cohort (Figures 5C and D in Multimedia Appendix 2), showing a tendency to underestimate risk in the new population. This suggests that while the model is effective for risk ranking, it may require recalibration using local data to optimize absolute probability estimates for different clinical settings.

## Appendix

Multimedia Appendix 1 Additional tables.

Multimedia Appendix 2 Additional figures.

## Abbreviations

AMI: acute myocardial infarction
AUC: area under the curve
BIDMC: Beth Israel Deaconess Medical Center
CBC: complete blood count
CK-MB: creatine kinase isoenzymes
CVD: cardiovascular disease
DCA: decision curve analysis
HR: hazard ratio
ICD-10: International Statistical Classification of Diseases, Tenth Revision
ICD-9: International Classification of Diseases, Ninth Revision
ICU: intensive care unit
K-M: Kaplan-Meier
LMR: lymphocyte-to-monocyte ratio
MIMIC-IV: Medical Information Mart for Intensive Care IV
NLR: neutrophil-to-lymphocyte ratio
NPR: neutrophil-to-platelet ratio
PIV: prognostic inflammatory value
PLR: platelet-to-lymphocyte ratio
RCS: restricted cubic spline
STROBE: Strengthening the Reporting of Observational Studies in Epidemiology
VIF: variance inflation factor

## Data Availability

The datasets generated and/or analyzed during this study are available from the following sources. The Medical Information Mart for Intensive Care IV (MIMIC-IV) database is publicly available via PhysioNet [30], with access requiring completion of the Collaborative Institutional Training Initiative (CITI) Data or Specimens Only Research training and approval by the review board. The dataset from Zhongnan Hospital is not publicly available due to patient privacy and ethical restrictions; however, deidentified data may be available from the corresponding author upon reasonable request and with permission from the Ethics Committee of Zhongnan Hospital. To facilitate reproducibility, the code has been deposited in a public repository and is available at gitee [31].
