# Correction: Complete Blood Count–Derived Inflammation Indices to Predict 3-Year All-Cause Mortality in Patients With Diabetes and Acute Myocardial Infarction in Critical Care: Retrospective Cohort Study With Single-Center External Validation

**DOI:** 10.2196/96740

**Published:** 2026-05-05

**Authors:** Tong Zhou, Kun Yang, Ying Yang, Song-Mei Liu

**Affiliations:** 1 Department of Clinical Laboratory Center for Gene Diagnosis and Program of Clinical Laboratory Zhongnan Hospital of Wuhan University Wuhan, Hubei China; 2 Department of Pharmaceutical Sciences Zhongnan Hospital of Wuhan University Wuhan, Hubei China

In “Complete Blood Count–Derived Inflammation Indices to Predict 3-Year All-Cause Mortality in Patients With Diabetes and Acute Myocardial Infarction in Critical Care: Retrospective Cohort Study With Single-Center External Validation” [1], the authors made one correction.

In the originally published manuscript, the affiliation for all authors was listed as:

Zhongnan Hospital of Wuhan University, Wuhan, China

The affiliation has been revised and now includes two departments, as follows:

Tong Zhou^1^, MS; Kun Yang^2^, PhD; Ying Yang^1^, PhD; Song-Mei Liu^1^, Prof Dr

^1^Department of Clinical Laboratory, Center for Gene Diagnosis and Program of Clinical Laboratory, Zhongnan Hospital of Wuhan University, Wuhan, Hubei, China

^2^Department of Pharmaceutical Sciences, Zhongnan Hospital of Wuhan University, Wuhan, Hubei, China

The correction will appear in the online version of the paper on the JMIR Publications website, together with the publication of this correction notice. Because this was made after submission to PubMed, PubMed Central, and other full-text repositories, the corrected article has also been resubmitted to those repositories.
