# Supplementary material for: Complete Blood Count–Derived Inflammation Indices to Predict 3-Year All-Cause Mortality in Patients With Diabetes and Acute Myocardial Infarction in Critical Care: Retrospective Cohort Study With Single-Center External Validation
Source: JMIR Med Inform. 2026 Mar 12;14:e83328. doi: 10.2196/83328 (PMC13022547; doi:10.2196/83328)
Supplement: Multimedia Appendix 1 [file medinform_v14i1e83328_app1.docx]

**Table S1.** Variance inflation factor (VIF) analysis and exclusion.

| Feature | vif_values |
| --- | --- |
| MCH | 355·0277428 |
| Hemoglobin | 351·5571332 |
| Hematocrit | 311·1611001 |
| MCV | 289·2328723 |
| RBC | 123·6438667 |
| MCHC | 113·4384558 |
| RDW SD | 44·58596947 |
| RDW | 37·23902008 |
| Bilirubin | 37·20614965 |
| I | 36·14634213 |
| Blood Gas pCO2 | 15·57860332 |
| Blood Gas pH | 15·564528 |
| Blood Gas Calculated Total CO2 | 14·19993813 |
| Chloride | 13·22542912 |
| Lymphocytes | 11·25095917 |
| Neutrophils | 11·17141235 |
| Sodium | 10·29782923 |
| Bicarbonate | 9·387170409 |
| Weight | 8·13548257 |
| Blood Gas Hematocrit Calculated | 7·530859139 |
| Blood Gas Hemoglobin | 7·276474749 |
| Anion Gap | 6·98766404 |
| BMI | 6·88096247 |
| AST | 5·095798488 |
| NLR | 4·873137501 |
| PLR | 4·731301188 |
| CABG | 3·89709713 |
| Monocytes | 3·699178491 |
| ALT | 3·583899835 |
| Urea Nitrogen | 3·515202429 |
| Creatinine | 3·479194201 |
| CK-MB | 2·724923183 |
| Blood Gas Base Excess | 2·707617006 |
| WBC | 2·684160317 |
| Troponin T | 2·528486272 |
| NPR | 2·521447069 |
| Albumin | 2·520594195 |
| Blood Gas Lactate | 2·445944939 |
| LMR | 2·406658307 |
| Blood Gas pO2 | 2·359332775 |
| Blood Gas Free Calcium | 2·284967105 |
| Blood Gas Sodium Whole Blood | 2·190199339 |
| Blood Gas Glucose | 2·166332746 |
| Potassium | 2·165782426 |
| PIV | 2·143918715 |
| Glucose | 2·134326531 |
| Calcium | 2·115806533 |
| gender | 2·040329334 |
| Fibrinogen Functional | 1·996865926 |
| Phosphate | 1·992941086 |
| NBPd | 1·824844843 |
| LD | 1·817458961 |
| Eosinophils | 1·777468756 |
| NBPs | 1·713484971 |
| Hypertension | 1·608821565 |
| Blood Gas Potassium Whole Blood | 1·604483143 |
| Blood Gas Oxygen Saturation | 1·58996485 |
| Age | 1·586706499 |
| Heart Failure | 1·583560266 |
| Urine pH | 1·583488598 |
| Immature Granulocytes | 1·579414844 |
| Magnesium | 1·52881844 |
| H | 1·462285105 |
| HR | 1·456997151 |
| PCI | 1·438578941 |
| Basophils | 1·431754758 |
| PT | 1·429115316 |
| RR | 1·393934259 |
| Alkaline Phosphatase | 1·366933086 |
| Urine Specific Gravity | 1·301703355 |
| Stroke | 1·288939665 |
| PTT | 1·239264062 |
| Hyperlipidemia | 1·232318165 |
| Insulin | 1·227218749 |
| MDS | 1·19881647 |
| Atrial Fibrillation | 1·157427157 |

**Table S2.** Comparison of baseline characteristics between survivors and non-survivors in the external dataset.

| **Variables** | **Total (n=166)** | **Survivor (n=114)** | **Non-survivor (n=52)** | ***P*** |
| --- | --- | --- | --- | --- |
| WBC (10^9/L) | 9·3 ± 4·1 | 8·6 ± 3·6 | 10·9 ± 4·8 | **.001** |
| RDW (%) | 13·9 ± 4·4 | 14·0 ± 5·3 | 13·8 ± 1·3 | .82 |
| Hemoglobin (g/dL) | 122·6 ± 25·1 | 125·6 ± 25·5 | 116·0 ± 23·1 | **.022** |
| Platelet Count (10^9/L) | 213·3 ± 76·9 | 225·3 ± 76·7 | 186·9 ± 71·0 | **.003** |
| Neutrophil Count (10^9/L) | 7·3 ± 4·0 | 6·4 ± 3·4 | 9·2 ± 4·6 | **<.001** |
| Lymphocyte Count (10^9/L) | 1·3 ± 0·6 | 1·4 ± 0·6 | 0·9 ± 0·5 | **<.001** |
| Monocyte Count (10^9/L) | 0·6 ± 0·3 | 0·6 ± 0·3 | 0·7 ± 0·4 | **.020** |
| Eosinophil Count (10^9/L) | 0·1 ± 0·1 | 0·1 ± 0·1 | 0·1 ± 0·1 | **.012** |
| Basophil Count (10^9/L) | 0·0 ± 0·0 | 0·0 ± 0·0 | 0·0 ± 0·0 | .56 |
| Hematocrit (%) | 37·1 ± 7·2 | 38·0 ± 7·1 | 35·3 ± 7·1 | **.025** |
| MCV (fL) | 91·1 ± 5·2 | 90·4 ± 5·2 | 92·5 ± 5·2 | **.016** |
| MCH (pg) | 30·0 ± 2·3 | 29·8 ± 2·4 | 30·4 ± 1·9 | .12 |
| MCHC (g/dL) | 329·2 ± 13·6 | 329·5 ± 15·0 | 328·6 ± 10·0 | .69 |

**Table S3.** Cox regression model of inflammatory related index.

| **Index** | **Groups** | **Non-adjusted** | | **Model 1** | | **Model 2** | |
| --- | --- | --- | --- | --- | --- | --- | --- |
|  |  | **HR (95% CI)** | ***P*-Value** | **HR (95% CI)** | ***P*-Value** | **HR (95% CI)** | ***P*-Value** |
| **LMR** |  |  |  |  |  |  |  |
|  | Quartile 1  (<1.17; N=205 ) | Reference |  | Reference |  | Reference |  |
|  | Quartile 2  (1.17-2.48; N=204) | 0.47 (0.35-0.62) | *<.001* | 0.48 (0.36- 0.64) | *<.001* | 0.71 (0.53-0.97) | *.03* |
|  | Quartile 3  (2.48-4.60; N=204) | 0.25 (0.18-0.35) | *<.001* | 0.29 (0.21- 0.41) | *<.001* | 0.65 (0.45-0.94) | *.02* |
|  | Quartile 4  (>4.60; N=205) | 0.10 (0.06-0.15) | *<.001* | 0.12 (0.07-0.19) | *<.001* | 0.44 (0.25-0.77) | *.004* |
| **NPR** |  |  |  |  |  |  |  |
|  | Quartile 1  (<0.033; N=208) | Reference |  | Reference |  | Reference |  |
|  | Quartile 2  (0.033-0.046; N=208) | 1.12 (0.78-1.62) | *.53* | 1.20 (0.83-1.74) | *.31* | 1.09 (0.74-1.59) | *.673* |
|  | Quartile 3  (0.046-0.064; N=208 ) | 1.10 (0.76-1.59) | *.614* | 1.15 (0.79-1.67) | *.46* | 1.20 (0.82-1.77) | *.351* |
|  | Quartile 4  (>0.064; N=208) | 1.93 (1.38-2.70) | *<.001* | 1.92 (1.37-2.70) | *<.001* | 1.35 (0.94-1.94) | *.104* |
| **PLR** |  |  |  |  |  |  |  |
|  | Quartile 1  (<83.50; N=208) | Reference |  | Reference |  | Reference |  |
|  | Quartile 2  (83.50-134.37; N=208) | 1.22 (0.81-1.84) | *.335* | 1.14 (0.75-1.72) | *.53* | 1.09 (0.71-1.66) | *.702* |
|  | Quartile 3  (134.37-225.65; N=208) | 1.83 (1.24-2.68) | *.002* | 1.71 (1.17-2.51) | *.006* | 1.22 (0.82-1.83) | *.328* |
|  | Quartile 4  (>225.65; N=208) | 3.35 (2.34-4.78) | *<.001* | 2.86 (1.99-4.10) | *<.001* | 1.39 (0.95-2.04) | *.088* |
| **NLR** |  |  |  |  |  |  |  |
|  | Quartile 1  (<3.50; N=208) | Reference |  | Reference |  | Reference |  |
|  | Quartile 2  (3.50-5.86; N=208) | 1.37 (0.90-2.10) | *.145* | 1.30 (0.85-1.99) | *.283* | 1.52 (0.98-2.35) | *.006* |
|  | Quartile 3  (5.86-10.62; N=207) | 1.98 (1.33-2.97) | *<.001* | 1.79 (1.19-2.69) | *.005* | 1.46 (0.86-2.22) | *.080* |
|  | Quartile 4  (>10.62; N=208) | 4.20 (2.90-6.08) | *<.001* | 3.69 (2.54-5.39) | *<.001* | 1.78 (1.19-2.65) | *.004* |
| **PIV** |  |  |  |  |  |  |  |
|  | Quartile 1  (<550.46; N=205) | Reference |  | Reference |  | Reference |  |
|  | Quartile 2  (550.46-1207.40; N=205) | 1.31 (0.76-1.68) | *.538* | 1.17 (0.79-1.73) | *.446* | 1.08 (0.72-1.62) | *.698* |
|  | Quartile 3  (1207.40-2619.32; N=204) | 1.51 (1.04-2.20) | *.03* | 1.60 (1.10-2.34) | *.014* | 1.33 (0.90-1.97) | *.148* |
|  | Quartile 4  (>2619.32; N=205) | 2.61 (1.84-3.69) | *<.0001* | 2.70 (1.90-3.84) | *<.0001* | 1.59 (1.09-2.30) | *.014* |

Model 1: Age, Gender, BMI; Model 2: Age, Gender, BMI, Troponin.T, AST, CK-MB, Insulin, PCI, CABG, Hyperlipidemia, Atrial fibrillation, Strok.

**Table S4.** Comprehensive performance comparison between the Stacked Model and LMR models including NRI, IDI, and goodness-of-fit metrics.

|  | Internal Validation | | External Validation | |
| --- | --- | --- | --- | --- |
|  | Value (95% CI) | P-value | Value (95% CI) | P-value |
| NRI (Categorical) | 0.005 (-0.093, 0.103) | 0.923 | 0.107 (-0.043, 0.257) | .16 |
| NRI (Continuous) | -0.228 (-0.463, -0.007) | 0.057 | -0.018 (-0.156, 0.120) | .80 |
| IDI | -0.019 (-0.054, 0.016) | 0.294 | -0.024 (-0.048, -0.001) | .055 |
