# Supplementary material for: Complete Blood Count–Derived Inflammation Indices to Predict 3-Year All-Cause Mortality in Patients With Diabetes and Acute Myocardial Infarction in Critical Care: Retrospective Cohort Study With Single-Center External Validation
Source: JMIR Med Inform. 2026 Mar 12;14:e83328. doi: 10.2196/83328 (PMC13022547; doi:10.2196/83328)
Supplement: Multimedia Appendix 2 [file medinform_v14i1e83328_app2.docx]

**Figure S1.** Forest plots for subgroup analyses of LMR, NPR and PLR with 3-year mortality.

**Figure S2.** Forest plots for subgroup analyses of NLR and PIV with 3-year mortality.


**Figure S3.** Comparison of prognostic performance by C-index.

**Figure S4.** Feature selection based on the Boruta algorithm. The horizontal axis represents the name of each variable, and the vertical axis represents the Z value of each variable. The green boxes represent important variables, and the red boxes represent unimportant variables.

**Figure S5.** Assessment of model performance.

(A-B) Decision Curve Analysis (DCA) for the validation set (A) and external validation set (B), showing the net benefit of the model at different threshold probabilities.

(C-D) Calibration plots for the validation set (C) and external validation set (D). The diagonal line represents perfect calibration; the dotted line represents the model's performance. Brier scores and Slopes are reported to quantify calibration accuracy.

**Figure S6.** Performance evaluation of the "Immune Indices + Vitals" model in the external validation cohort.

To address the real-world applicability of the model, a leaner model combining inflammatory indices with readily available vital signs was validated in the external cohort.

(A) Calibration Plot. The plot assesses the agreement between the predicted and observed 3-year mortality risks. The gray diagonal band represents perfect calibration (Ideal). The solid black line indicates the logistic calibration of the model, while the dotted line represents the nonparametric fit. The vertical spikes (rug plot) along the x-axis depict the distribution of predicted probabilities. The Brier score (0.167) and Slope (1.334) suggest acceptable calibration in the external setting.

(B) DCA. The curve illustrates the clinical utility of the model. The y-axis measures the net benefit. The purple line represents the "Vitals + Immune indices" model. This model demonstrates a higher net benefit compared to the default strategies of "Treat All" (red line) and "Treat None" (green line) across a reasonable range of threshold probabilities.

**Figure S7.** Performance evaluation of the simple benchmark model (Age + Albumin + Creatinine).

To evaluate the incremental value of the inflammatory indices, a benchmark model comprising only Age, Albumin, and Creatinine was constructed and validated.

(A-B) Calibration Plots. The plots display the calibration of the benchmark model in the internal validation set (A) and the external validation set (B). While the model showed acceptable calibration internally (C-index: 0.800), it exhibited poor generalizability and calibration in the external cohort (C-index: 0.542; Slope: 0.024), indicating a lack of predictive accuracy across different centers.

(C-D) DCA. The curves illustrate the net benefit of the benchmark model in the internal (C) and external (D) validation sets. The purple line (in C) and cyan line (in D) represent the benchmark model. The model provided limited net benefit in the external cohort compared to the "Treat None" strategy, further highlighting the necessity of integrating inflammatory indices for robust risk stratification.

**Figure S8.** Calibration curves of the single model (LMR) for assessing goodness-of-fit.

(A-B) Display the calibration plots for the LMR model in the internal validation cohort and external validation cohort, respectively. The x-axis represents the predicted probability, and the y-axis represents the actual probability. The diagonal gray line represents perfect prediction (ideal model). The solid black line indicates the performance of the LMR model (logistic calibration), and the dotted curve represents the nonparametric fit. The histograms (spikes) along the x-axis depict the distribution of predicted probabilities. Brier scores and slopes are reported to further evaluate calibration accuracy.


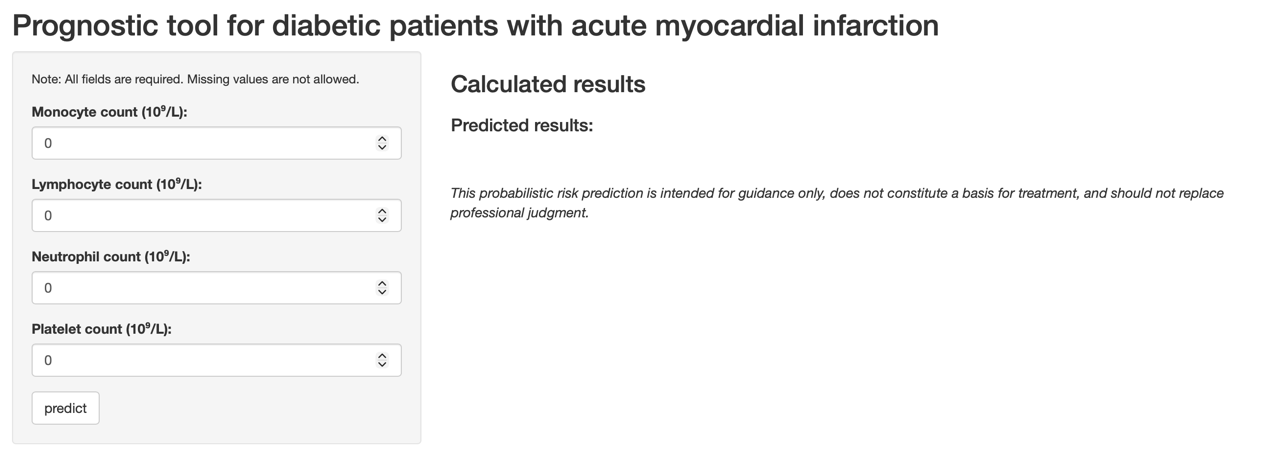


**Figure S9.** Updated interface of the web tool.
